# Sound velocity of hexagonal close-packed iron to the Earth’s inner core pressure

**DOI:** 10.1038/s41467-022-34789-2

**Published:** 2022-11-25

**Authors:** Daijo Ikuta, Eiji Ohtani, Hiroshi Fukui, Takeshi Sakai, Daisuke Ishikawa, Alfred Q. R. Baron

**Affiliations:** 1 Department of Earth Science, Tohoku University, Sendai, Miyagi 980-8578 Japan; 2 Materials Dynamics Laboratory, RIKEN SPring-8 Center, Sayo, Hyogo 679-5148 Japan; 3 Japan Synchrotron Radiation Research Institute, Sayo, Hyogo 679-5198 Japan; 4 Geodynamics Research Center, Ehime University, Matsuyama, Ehime 790-8577 Japan

**Keywords:** Solid Earth sciences, Core processes, Geophysics

## Abstract

Here we determine the compressional and shear wave velocities (*v*_p_ and *v*_s_) of hexagonal close-packed iron, a candidate for the main constituent of the Earth’s inner core, to pressures above 300 gigapascals using a newly designed diamond anvil cell and inelastic X-ray scattering combined with X-ray diffraction. The present results reveal that the *v*_p_ and *v*_s_ of the Preliminary reference Earth model (PREM) inner core are 4(±2)% and 36(±17)% slower than those of the pure iron, respectively at the centre of the core. The density and sound velocity of the PREM inner core can be explained by addition of 3(±1) wt% silicon and 3(±2) wt% sulphur to iron‒5 wt% nickel alloy. Our suggested inner core composition is consistent with the existing outer core model with oxygen, as the growth of the inner core may have created a secular enrichment of the element in the outer core.

## Abstract (web summary)

New constraints on the composition of Earth’s inner core are provided by experimental verification of Birch’s law for hexagonal close-packed iron to pressure above 300 gigapascals, about double the pressure achieved in previous investigations

## Introduction

Earth’s thermal history can be understood from a perspective of timing and nature of inner core crystallisation. The properties of iron under extreme pressure are of great importance to understand the Earth’s inner core. Comparison of the density of hexagonal close-packed (hcp) iron under the core conditions and seismic models, e.g., the Preliminary reference Earth model (PREM)^1^ indicates that the inner core is less dense than pure iron suggesting it may contain some light elements^2–4^. The most well-known physical property of the core is the sound velocity, specifically the compressional (*v*_p_) and shear (*v*_s_) wave velocities, derived from seismological observations. However, the sound velocity of pure hcp-iron under core conditions remains debated as there are no experimental results at static pressures comparable to the inner core due to the difficulties in measuring sound velocity under such high-pressure conditions. Thus, the discussion about core properties has had to rely on extrapolations from data at lower pressures^5–11^. Different methods have been proposed to extrapolate the relation between sound velocity and density, including a linear relation, known as Birch’s law^2^, or a power-law relation^7^. Such differences in extrapolation method contribute to the large deviation reported at high pressures when the sound velocity measurements were limited to a low pressure^7,9,11^. In order to solve this problem, it is indispensable to directly determine the sound velocity under the inner core density and pressure without extrapolation.

The Earth’s inner core has a density, *ρ*, of 12.8‒13.1 g cm^−3^ at pressures varying from 330 GPa (inner core boundary, ICB) to 365 GPa (centre of the Earth/core, COE)^1^. Here, we measured *v*_p_ of hcp-iron to a *ρ* of 13.87 g cm^−3^ exceeding the inner core density. The corresponding pressure is 310‒327 GPa^3,4,12^ depending on the pressure scale. This is the highest static pressure so far achieved for inelastic X-ray scattering (IXS) and in situ X-ray diffraction (XRD) methods at BL43LXU^13^ of the RIKEN SPring-8 Center, and indeed with any static measurement of a sound velocity. It was made possible by taking advantage of several developments including an improved design of the diamond anvil cell using a “stepped-bevel” anvil (Supplementary Fig. 1) (similar to Fei et al.^4^ or Dewaele et al.^14^) combined with highly optimised X-ray optics with a 5 μm beam size, and special Soller screens^15^ specifically optimised to improve the signal to noise ratio for this work. These allowed us to extend the accessible pressure range to that of the inner core. We were also aided by the diamond “cupping” that increased the sample thickness and gave more nearly hydrostatic pressure above 300 GPa (see Methods and Supplementary Figs. 2‒3). Based on the measurement of *v*_p_ of hcp-iron at the core pressures, we evaluated the velocity deficits between hcp-iron and the PREM inner core and consider a possible candidate light element composition of the inner core to account for both the *v*_p_ and *v*_s_ of the PREM.

## Results

### Sound velocity measurement by inelastic X-ray scattering

**Fig. 1:**
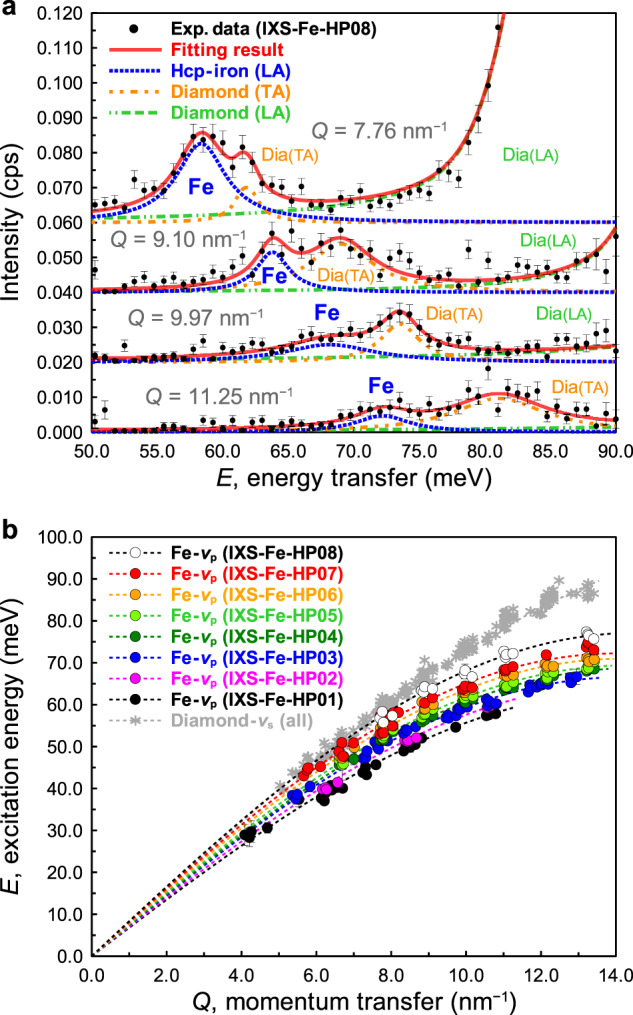
Inelastic X-ray scattering (IXS) spectra and dispersion curves of hexagonal close-packed (hcp) iron. **a** IXS spectra of hcp-iron at 13.87 g cm^−3^ (corresponding to 310 GPa^3^) for different momentum transfers (*Q* = 7.76, 9.10, 9.97, and 11.25 nm^−1^) at ambient temperature by an offset of 0.02 cps in the vertical axis for clarity. The data were fit with peaks for longitudinal acoustic (LA) mode of hcp-iron (blue), the transverse acoustic (TA) and LA mode of diamond (orange and green). **b** The dispersion curves of hcp-iron determined at pressures from 154 to 310 GPa based on the equation of state of hcp-iron^3^. The fitted results were given in Table 1. The data for momentum transfer *Q* and energy transfer *E* of TA mode of diamond is plotted as grey asterisks. The error bars represent one standard deviation (1σ) uncertainties and are mostly smaller than the circles.

Figure 1a shows spectra collected at the highest *ρ* of 13.87 g cm^−3^ corresponding to 310‒327 GPa^3,12^. We identified the IXS peaks for the longitudinal acoustic (LA) mode of hcp-iron and the transverse acoustic (TA) and LA modes of diamond together with an elastic peak (not shown). At the highest pressures, the IXS contributions from the diamond TA mode were close to those of the sample LA mode, thus, we used a Soller screen modified to improve the intensity of the sample LA mode relative to the diamond TA mode. While this reduced the signal to the level of 0.01 s^−1^ for the sample peaks, the signal to noise ratio improved, thus, the contributions could be separated by peak fitting. The phonon dispersion curves of all experimental conditions are shown in Fig. 1b and numerical values are given in Table 1. Typical two-dimensional XRD image and integrated XRD patterns are shown in Supplementary Fig. 4.

### Sound velocity of hexagonal close-packed iron

**Fig. 2:**
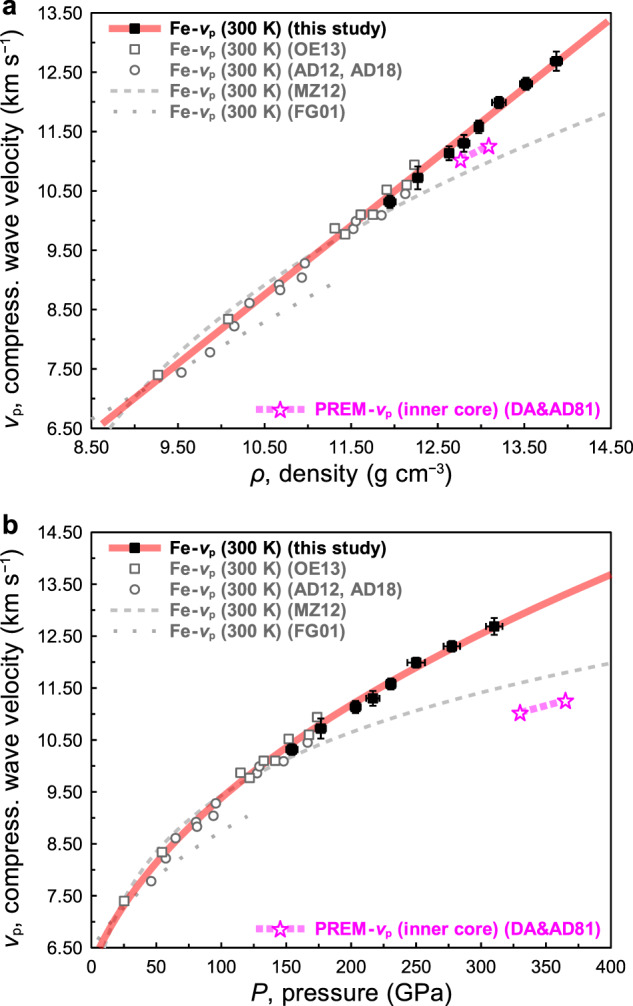
Compressional wave velocity (*v*_p_) of hexagonal close-packed (hcp) iron at high pressure. **a** The *v*_p_‒*ρ* relation of hcp-iron at ambient temperature. Solid squares are the results of this work; open squares from Ohtani et al. (OE13)^8^; circles from Antonangeli et al. (AD12, AD18)^6,10^; a dashed curve extrapolated by a power-law relation from Mao et al. (MZ12)^7^; and a dotted curve from Fiquet et al. (FG01)^5,11^. The magenta dashed curve with stars is the Preliminary reference Earth model (PREM) inner core (DA&AD81)^1^. **b** The pressure dependency of *v*_p_ of hcp-iron at ambient temperature. The pressures were evaluated by the equation of state of hcp-iron by Dewaele et al.^3^ The symbols are the same as those in Fig. 2a. The error bars represent 1σ uncertainties.

The *v*_p_ of hcp-iron determined in this work are plotted with previous IXS results at lower pressures and ambient temperature^5–8,10^ in Fig. 2a. It is clear that a linear *v*_p_‒*ρ* relation, Birch’s law^2^, is valid for hcp-iron up to 13.87 g cm^−3^, which covers inner core density regions^1^ of 12.8‒13.1 g cm^−3^. We find *v*_p_ (m s^−1^) = 1.162(±0.015) *ρ* (kg m^−3^) − 3450(±166) (m s^−1^) by combining the present results and those of previous IXS results^6,8,10^. The *v*_p_ of hcp-iron at 12.8‒13.1 g cm^−3^ and ambient temperature is 11.4‒11.8 km s^−1^, which is ~3‒4% faster than that of PREM inner core. Figure 2b shows the pressure dependence of *v*_p_ of hcp-iron using pressures determined by the equation of state (EoS) by Dewaele et al.^3^. The density, *ρ*, of hcp-iron at the inner core pressure (330‒365 GPa) and ambient temperature is estimated to be 14.1‒14.4 g cm^−3^, which is 9‒10% larger than that of the PREM inner core of 12.8‒13.1 g cm^−3^. As determined from our work, *v*_p_ of hcp-iron at inner core pressure (330‒365 GPa) and ambient temperature is 12.7‒13.3 km s^−1^, which is significantly faster (~15‒18%) than that of the PREM inner core. The present sound velocity measurements of hcp-iron to the core density of 13.87 g cm^−3^ showed a linear *v*_p_‒*ρ* relation is good for the extrapolation of *v*_p_ of hcp-iron^6–8^ to the PREM inner core density. In fact, given the extended data range now available, extrapolations both by a linear and exponential relations provide similar values of the sound velocity for the inner core density, eliminating the previous contradictions originating from different extrapolation methods. This shows the importance of performing measurements to the inner core density.

**Fig. 3:**
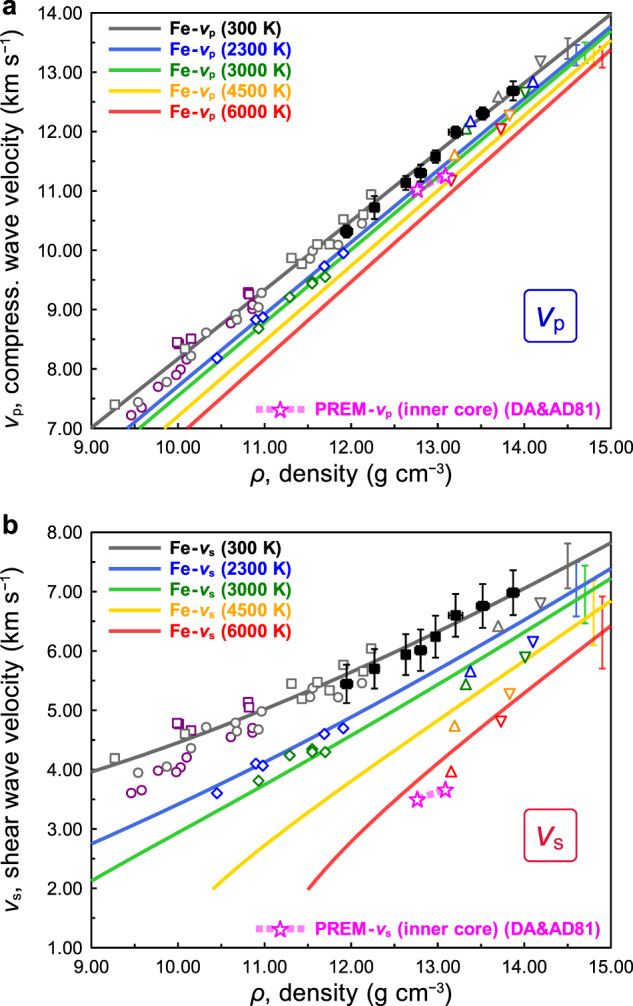
Compressional (*v*_p_) and shear (*v*_s_) wave velocities of hexagonal close-packed (iron) at high pressure and temperature. **a** The *v*_p_‒*ρ* relation, i.e., Birch’s law^2^, for hcp-iron at different temperature. We include the ambient temperature data of this work (black solid squares) and Ohtani et al. (grey open squares)^8^, Antonangeli et al. (grey circles)^6,10^, and high temperature data by Sakamaki et al. (blue diamonds: 2300 K, green diamonds: 3000 K)^16^, Ohtani et al. (purple squares: 400‒1000 K)^8^ and Antonangeli et al. (purple circles: 700‒1110 K)^6^ for deriving the temperature dependence. The theoretical data (coloured open triangles, grey: 0 K, blue: 2300 K, green 3000 K, orange: 4500 K, and red: 5500 K)^17,18^ and the Preliminary reference Earth model (PREM) inner core (a magenta curve with stars, DA&AD81)^1^ are shown for comparison. Coloured lines represent the *v*_p_‒*ρ* relations derived from the experimental data of this work with other inelastic X-ray scattering (IXS) experimental data^6,8,10,16^ (grey: 300 K, blue: 2300 K, green 3000 K, orange: 4500 K, and red: 6000 K). **b** The *v*_s_‒*ρ* relation at different temperatures. The calculation of the *v*_s_ for the IXS experiments are given in the Methods. The curves showing the *v*_s_–*ρ* relations are derived from the linear *v*_p_‒*ρ* relations in Fig. 3a and the equation of state^3^. Symbols given in this figure are the same as those of Fig. 3a. The error bars represent 1σ uncertainties.

We parameterise the temperature dependence^16^ of the Birch’s law as:

1 $${v}_{{{{{{\rm{p}}}}}}}=A\rho -B-C(T-{T}_{0})\,(\rho_{{{\rm{x}}}}-\rho ),$$

where *A*, *B*, *C*, and *ρ*_x_ are the parameters of the relation, *T* is temperature, and *T*_0_ is ambient temperature, i.e., 300 K. The effect of temperature on the *v*_p_‒*ρ* relation, i.e., the Birch’s law, of hcp-iron at high temperature is re-evaluated by combining the present results with previous measurements at high temperature^6,8,16^. This is shown in Fig. 3a. We find the updated parameters, *A* = 1.162(±0.015) m^4^ kg^−1^ s^−1^, *B* = 3450(±166) m s^−1^, *C* = 2.5(±0.2) × 10^−5^ m^4^ kg^−1^ s^−1^ K^−1^, and *ρ*_X_ = 19.2(±0.3) × 10^3 ^kg m^−3^. Our results for the *v*_p_‒*ρ* relation at high temperature are consistent with the ab initio calculations made by Vocadlo et al.^17^ and Martorell et al.^18^: their theoretical results provide slightly higher *v*_p_ at the inner core conditions, but still within the uncertainty of our estimation.

The *v*_s_‒*ρ* relation of hcp-iron is shown in Fig. 3b, where *v*_s_ is related to *v*_p_ and the adiabatic bulk modulus (*K*_*S*_) as follows:

2 $${K}_{S}=\rho \left({v}_{{{{{{\rm{p}}}}}}}^{2}-\frac{4}{3}{v}_{{{{{{\rm{s}}}}}}}^{2}\right).$$

The details of estimation of *v*_s_ are given in Methods. There are discrepancies in *v*_p_‒*ρ* and *v*_s_‒*ρ* relations of hcp-iron among ab initio calculations^17–19^ as discussed by Bouchert et al.^19^. Our result is generally consistent with calculations by Vocadlo et al.^17^ and Martorell et al.^18^.

**Fig. 4:**
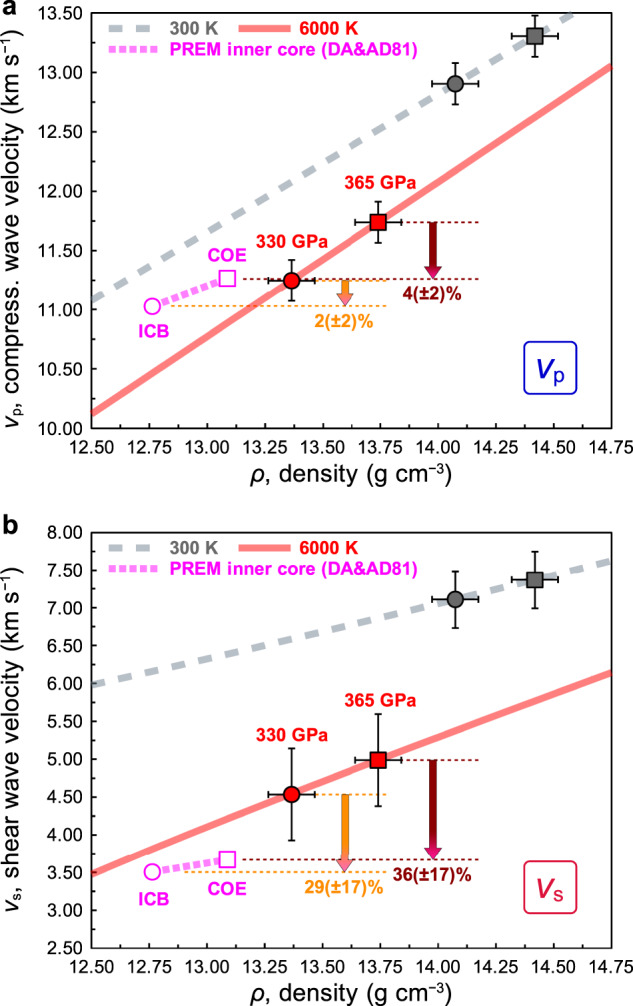
Comparison of density‒velocity relations of hexagonal close-packed (hcp) iron at inner core conditions with Preliminary reference Earth model (PREM). The *v*_p_ deficit (**a**) and the *v*_s_ deficit (**b**) in the inner core. The *v*_p_ and *v*_s_ of the PREM inner core (DA&AD81)^1^ compared to those of hcp-iron at ambient temperature (grey dashed curves) and 6000 K (red curves: fitted *v*_p_‒*ρ* and *v*_s_‒*ρ* relations) at 330 GPa (inner core boundary, ICB, and circle symbols) and 365 GPa (centre of the Earth/core, COE, and square symbols). The equation of state of hcp-iron by Dewaele et al.^3^ was used for the calculation. The *v*_p_ deficit and the *v*_s_ deficit are 2(±2)% and 29(±17)% at the ICB, and 4(±2)% and 36(±17)% at the COE, respectively. The error bars represent 1σ uncertainties.

Figure 4 shows the *v*_p_‒*ρ* and *v*_s_‒*ρ* relations for hcp-iron at ICB and COE at 300 K and 6000 K based on the EoS of Dewaele et al.^3^ compared to the values of the PREM inner core^1^. Pure hcp-iron has 2(±2)% higher *v*_p_ and 29(±17)% higher *v*_s_ at the ICB, and 4(±2)% higher *v*_p_ and 36(±17)% higher *v*_s_ at the COE, respectively, compared to those of the PREM inner core with a typical estimated inner core temperature of 6000 K (e.g., refs. 20–22). The present *v*_p_ deficit is smaller than that estimated previously by Sakamaki et al.^16^. A large *v*_s_ deficit compared to the *v*_p_ deficit of the PREM inner core relative to hcp-iron is an important constraint on the sound velocity deficits determined by direct static measurements at core densities. This provides a strong constraint for estimation of the light element contents in the inner core.

## Discussion

The inner core *v*_p_ and *v*_s_ deficits reported in this work together with the density deficit^3^ of the PREM inner core compared to pure hcp-iron provides important constraints on the composition of the inner core. As pure iron cannot explain the density and velocity of the PREM, many studies on physical properties of iron alloys or iron compounds were performed to account for those deficits and concluded that light elements play a major role to account for the deficits^2–4^.

Geochemical studies^23^ have suggested silicon and sulphur may be the major light elements in the core. A numerical study^24^ clarified the partitioning behaviour of silicon, sulphur, and oxygen between solid and liquid iron, and revealed a strong partitioning of oxygen into the liquid outer core, indicating the silicon and sulphur bearing inner core with oxygen depletion. The high sound velocity of FeO suggests oxygen is not a major light element of the inner core (Supplementary Fig. 5). Therefore, a reasonable combination of light elements to account for the PREM inner core may be the mixture of the three-endmember components of iron (or iron‒nickel alloy), iron‒silicon alloy (e.g., Fe_0.89_Si_0.11_), and iron‒sulphur compound (e.g., Fe_3_S).

**Fig. 5:**
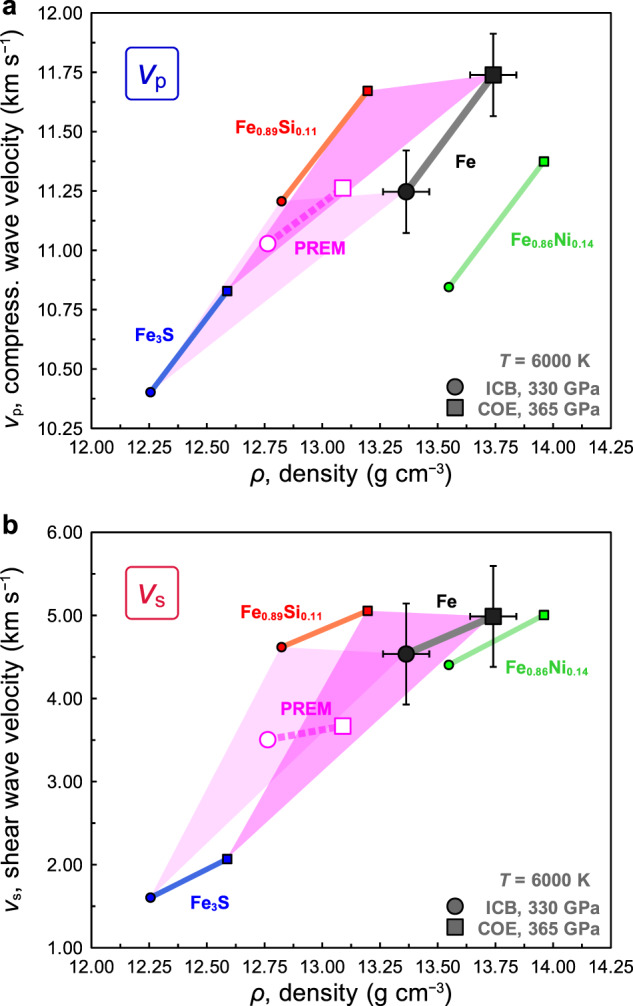
Comparison of *v*‒*ρ* relations of hexagonal close-packed (hcp) iron, iron alloys Fe_0.89_Si_0.11_ and Fe_0.86_Ni_0.14_, and iron sulphur compound Fe_3_S with Preliminary reference Earth model (PREM) at the inner core boundary (ICB, 330 GPa, and 6000 K) and centre of the Earth/core (COE, 365 GPa, and 6000 K) conditions. The *v*_p_‒*ρ* (**a**) and the *v*_s_‒*ρ* (**b**) relations of each material. The circle and square symbols indicate ICB and COE conditions, respectively. The coloured symbols are as follows: black is hcp-iron from this study (pressure is evaluated by the Dewaele et al.^3^), red is Fe_0.89_Si_0.11_ (6 wt% silicon)^25,26^, green is Fe_0.86_Ni_0.14_ (15 wt% nickel)^27,28^, blue is Fe_3_S^29,30^, and magenta (open symbol) is PREM^1^. The *v*‒*ρ* relations for hcp-iron at the ICB and COE conditions are determined by this work (see text and Methods). The *v*‒*ρ* relations of other iron alloys and iron compound are calculated from previous studies on sound velocity and equations of state^25–30^, and the present temperature dependence of hcp-iron (details are in Methods) for FeNi_0.15_ and Fe_3_S. The magenta triangles represent the potential *v*‒*ρ* relations of the iron compounds consisting of three endmembers (hcp-iron, Fe_0.89_Si_0.11_, and Fe_3_S) (lighter and darker colours indicate ICB and COE conditions, respectively). The error bars represent 1σ uncertainties for hcp-iron.

Figure 5 summaries the *v*_p_‒*ρ* (Fig. 5a) and *v*_s_‒*ρ* (Fig. 5b) relations of hcp-iron, iron alloys (Fe_0.89_Si_0.11_ and Fe_0.86_Ni_0.14_), and iron‒light element compounds (Fe_3_S) under the inner core conditions (330‒365 GPa, 6000 K). We derive the properties *v*_p_, *v*_s_, and *ρ* at ICB and COE using previous sound velocity measurements and EoSs^25–30^(details are given in Methods). The temperature dependence of *v*_p_ for Fe_0.89_Si_0.11_ was reported by Sakairi et al.^25^, and those of Fe_0.86_Ni_0.14_ and Fe_3_S are assumed to be the same as that of hcp-iron in this work.

The triangles of these endmember components at both ICB and COE in Fig. 5 (lighter and darker magenta colours indicate ICB and COE conditions, respectively) can account for *ρ*, *v*_p_, and *v*_s_ of the PREM inner core. The uncertainties associated with velocity measurements and the EoS of hcp-iron are shown in the figure. When we consider iron‒silicon‒sulphur alloy, *ρ*, *v*_p_, and *v*_s_ of the PREM inner core can be accounted for by addition of 4(±1) wt% silicon and 3(±1) wt% sulphur at ICB and 4(±1) wt% silicon and 4(±1) wt% sulphur at COE, assuming Fe_0.89_Si_0.11_ and Fe_3_S endmembers have the same uncertainties as hcp-iron. Assuming that the inner core is iron with a homogeneous distribution of light elements, the best estimation of the light elements in the whole inner core is 4(±1) wt% silicon and 3(±2) wt% sulphur. However, meteorite composition and geochemistry strongly suggest that the core contains a substantial amount of nickel^23^. When we consider the nickel content of the inner core has the chondritic Ni/Fe ratio (e.g., ref. 31) as 0.06, *ρ*, *v*_p_, and *v*_s_ of the PREM inner core can be accounted for by addition of 3(±1) wt% silicon and 3(±1) wt% sulphur at ICB and 2(±1) wt% silicon and 4(±1) wt% sulphur at COE to the metallic iron‒nickel‒silicon‒sulphur alloy (~5 wt% nickel, which is the chondritic Ni/Fe ratio). There may be some heterogeneities in the light element content between ICB and COE, but those are within our uncertainties. Assuming that the inner core has a homogeneous distribution of light elements with the chondritic Ni/Fe ratio, the best estimation of the light elements in the whole inner core then becomes 3(±1) wt% silicon and 3(±2) wt% sulphur.

Our estimations of the silicon and sulphur contents of the inner core are based on the deficits of compressional and shear velocities and density, whereas most previous estimations were based only on the density deficit of the inner core, and occasionally, the *v*_p_ deficit^10,32,33^. Therefore, our results are more definite compared to the previous estimations. Additionally, previous studies provided contradictory estimates in silicon and sulphur contents in the inner core, ~5‒8 wt%^34,35^ and ~1‒4 wt%^10,32,33^ in silicon content, whereas the sulphur content varies from 1.3 to 3.7 wt%^36,37^. The present analysis suggests that both silicon and sulphur may be needed to account for the properties of the inner core. McDonough^23^ estimated the amount of silicon and sulphur of the bulk core to be 6 wt% and 1.9 wt%, respectively, from geochemical constraints. The numerical study by Alfe et al.^24^ suggested existence of very small amount oxygen around 0.3 wt% (0.2 mol%), and silicon and/or sulphur around 4.5‒5 wt% (8.5 mol%) in the inner core to account for the density of the PREM inner core. The present mineral physics estimate of silicon and sulphur contents in the inner core, 3(±1) wt% silicon and 3(±2) wt% sulphur with ~5 wt% nickel, is consistent with the geochemical and numerical estimations^23,24,38^.

Martorell et al.^18^ introduced the pre-melting effect in hcp-iron to account for the *v*_p_ and *v*_s_ of the PREM inner core. However, our analysis indicates such an anomalous effect is not required and introduction of both silicon and sulphur components can explain the inner core deficit of *v*_p_, and *v*_s_. Although the solid‒liquid partitioning data of iron alloy is not available under the inner core conditions, previous data suggested very small value of partition coefficient (*D*) of oxygen [*D*(O) ~0, e.g., refs. 24,39] and sulphur [*D*(S) = 0.4‒0.8, e.g., refs. 39,40], and very weak partitioning of silicon between solid and liquid iron alloys [*D*(Si) ~1, e.g., refs. 41–43]. Therefore, present silicon and sulphur abundances in the inner core is consistent with the outer core containing a large amount of oxygen with a limited amount of sulphur and silicon by ab initio calculations^21,24,44^. The difference in the light element contents such as oxygen, sulphur, and silicon between inner and outer cores suggests enrichment of oxygen in the outer core during crystallisation of the inner core.

The *v*_p_‒*ρ* and *v*_s_‒*ρ* relations of hcp-iron, iron alloys (Fe_0.89_Si_0.11_, 6 wt% silicon alloy and Fe_0.86_Ni_0.14_, 15 wt% nickel alloy), and iron‒light element compounds (Fe_7_C_3_, Fe_3_C, Fe_3_S, FeH, and FeO) under the inner core conditions (330‒365 GPa, 6000 K) are summarised in Supplementary Fig. 5. We derive the properties *v*_p_, *v*_s_, and *ρ* at ICB and COE using previous sound velocity measurements and EoSs^25–30,45–50^ (details are given in Methods) and assuming a similar temperature dependence of *v*_p_ as that of hcp-iron determined above, except for Fe_0.89_Si_0.11_, Fe_7_C_3_, Fe_3_C, and FeO, which were determined experimentally^25,45,46,50^. It is not possible to rule out other compositions such as carbon^45,46,51^ and hydrogen^52–54^ since in most cases the uncertainties of estimations of *v*_p_ and *v*_s_ in the iron‒light element compounds are greater than those of the present results on pure hcp-iron due to large extrapolations. Recently, Wang et al.^53^ and He et al.^54^ suggested existence of the superionic iron‒light element compounds, including hydrogen to account for the large reduction of *v*_s_ of the inner core compared to hcp-iron. If we consider the existence of such superionic iron‒light element compounds, the alloying light elements in the inner core might contain hydrogen with smaller amounts of other light elements than those estimated here.

## Methods

### Starting material and high-pressure generation

The rhenium gasket material was a 250 μm thick foil, 99.97% purity, from Alfa Aesar, that was pre-compressed to a thickness of ~10 μm. A hole of 15 μm diameter was drilled for the sample chamber. Iron powder (99.99% purity from Wako Chemicals) was placed in the rhenium gasket hole without pressure medium. Double bevel diamond anvils with a culet size of 50 μm were used with a rhenium gasket to generate the pressure up to 250‒278 GPa^3,4,12^. Higher pressures were generated by the improved “stepped bevel” diamond anvil. The stepped bevel anvils with a culet size of 30 μm was made by machining a flat surface of 70 μm in diameter with a depth of ~3 μm outside the 30 μm culet of (100)-oriented single crystalline double bevel anvils, using a dual focused ion beam (FIB)‒scanning electron microscope (SEM) module (Scios). Supplementary Fig. 1a shows the shape of the anvil determined by the field emission SEM after the machining. Supplementary Fig. 1b shows the microscopic image under maximum pressure (310 GPa^3^, 315 GPa^4^, and 327 GPa^12^). The anvil obviously keeps the stepped bevel shape with the sample chamber of ~20 μm (expanded from the starting size of 15 μm), which is brightest area.

Supplementary Fig. 2a shows iron sample or rhenium gasket thickness distribution of stepped bevel anvil compared with a double bevel anvil at 278 GPa^3^ derived from X-ray absorption. For stepped bevel anvil, the shape of the sample is slightly more “cupped” compared to the double bevel anvil. Supplementary Fig. 2b shows the integrated XRD profiles at 278 GPa^3^ in sample position and 5, 10, 20, and 35 μm off-centred positions. Pressure decreases by ~10 GPa and ~20 GPa at 5 μm and 10 μm off-centred positions, respectively, a pressure distribution similar to the diamond anvil used in refs. 4,14. In ideal hcp-iron, 101 peak has maximum intensity, and 100 and 002 peaks have almost same intensities. However, in the XRD profile by a double bevel anvil, there is no obvious 002 peak, and 100 peak is stronger than 101 peak. This indicates the pressure generation by a double bevel anvil causes strong preferred orientation. Compared with the pressure generation by a double bevel anvil, XRD profiles by a stepped bevel anvil indicates more quasi-hydrostatic conditions, because there is 002 peak and 101 peak has maximum intensity. In high-pressure diamond anvil cell (DAC) experiments, the effect of uniaxial compression on preferred orientation should be considered^16,55–57^. Typical profiles of crystal axis concentrations are shown in Supplementary Fig. 3a by stereographic projection. Supplementary Fig. 3b shows the experimentally measured distributions of crystal direction (due to preferred orientation) at 278 GPa of both stepped bevel and double bevel anvils, with calculated *v*_p_ anisotropy from the theoretical data^17,18^. The experimental preferred orientation by a stepped bevel anvil shows weak preferred orientation and the angle between the *c*-axis and the momentum transfer is widely distributed, from 30 to 90 degrees. On the other hand, the experimental preferred orientation by a double bevel anvil shows strong preferred orientation and *c*-axis of hcp-structure is almost parallel with the compressional direction (and also incident X-ray beam). The effect of these experimental preferred orientations and calculated *v*_p_ anisotropy^17,18^ on the sound velocity measurement is shown in Supplementary Fig. 3c. The effects of anisotropy on *v*_p_ in stepped bevel anvil experiments and double bevel anvil experiments are <0.5% and <1.3%, respectively. The effect of anisotropy on *v*_p_ is within the uncertainty of the present *v*_p_ measurement shown in Table 1. The anisotropy is smaller in stepped bevel anvil experiments, although it is still small in double bevel anvil experiments. Therefore, our experimental *v*_p_ can be estimated to be almost consistent with the *v*_p_ with a random orientation.

**Table 1:** Experimental conditions and results

Run-no.	*ρ*, density (g cm^−3^)	*v*_p_, compress. wave velocity (km s^−1^)	*Q*_max_(*v*_p_) (nm^−1^)	*P*_(Dewaele, 2006)_ (GPa)^3^	*P*_(Fei, 2016)_ (GPa)^4^	*P*_(Smith, 2018)_ (GPa)^12^
IXS-Fe-HP01	11.95 (±0.07)	10.32 (±0.10)	14.7 (±0.5)	154	155	157
IXS-Fe-HP02	12.27 (±0.06)	10.72 (±0.19)	14.5 (±0.5)	177	177	181
IXS-Fe-HP03	12.63 (±0.05)	11.13 (±0.12)	14.3 (±0.4)	203	204	210
IXS-Fe-HP04	12.80 (±0.06)	11.30 (±0.14)	14.5 (±0.2)	217	218	224
IXS-Fe-HP05	12.97 (±0.05)	11.58 (±0.11)	14.3 (±0.2)	231	232	239
IXS-Fe-HP06	13.21 (±0.08)	11.99 (±0.10)	14.1 (±0.2)	250	252	261
IXS-Fe-HP07	13.52 (±0.07)	12.30 (±0.10)	14.1 (±0.2)	278	280	291
IXS-Fe-HP08	13.87 (±0.07)	12.69 (±0.16)	14.1 (±0.2)	310	314	327

### IXS‒XRD measurement

The *v*_p_ and density, *ρ*, of hcp-iron were measured using a DAC combined with IXS and XRD methods at BL43LXU^13^ of SPring-8. The Si (9 9 9) reflection at 17.79 keV provided a resolution of 2.8 meV. A fine focused beam of 5 μm × 5 μm by a multilayer Kirkpatrick-Baez mirror pair^15^ was employed for the IXS‒XRD measurements. The energy of the LA mode of hcp-iron, and the LA and TA modes of diamond from DAC were extracted by fitting with combination of Gaussian and Lorentzian functions, so called pseudo-Voigt function. It was difficult to separate the signal of the LA mode of the sample from the TA mode of diamond because the IXS signal from diamond was large compared to the sample in the DAC at pressures above 100 GPa. Thus, in order to reduce the background from diamond and to improve signal to noise ratio, we installed a Soller screen^15^ downstream of the DAC. For the higher density measurements presented here (>13.5 g cm^−3^), the Soller screen was modified from that described in ref. 15 in order to improve the signal-to-noise ratio. While this modification (reduction in the slit width) also reduced the signal, and therefore led to longer counting times, the trade-off was deemed acceptable to carry out the experiments. We note that results with different diamond anvil cell types and with different Soller screens were consistent. In particular, the IXS experiments at 278 GPa^3^ were performed in multiple configurations and the observed peak positions were consistent within the error as shown in Fig. 1b. The in situ XRD patterns of hcp-iron were collected under the same optical setup at BL43LXU beamline before and after the IXS measurements using a flat panel (FP) detector (C9732DK, Hamamatsu Photonics). The distance between the sample and the flat panel detector was calibrated with a cerium dioxide standard (NIST, National Institute of Standards and Technology). The XRD patterns were analysed using the software packages by Seto et al.^58,59^ and the lattice parameters, densities, and the information of preferred orientation for hcp-iron were determined from the XRD patterns (Supplementary Figs. 2‒4). The experimental pressure was evaluated by the EoS of hcp-iron^3,4,12^ (Table 1).

The relation between the excitation energy, *v*_p_ and the momentum transfer is given by

3 $$E=\frac{h{{v}_{{{{{{\rm{p}}}}}}}Q}_{{{\max }}}}{{\pi }^{2}}{{\sin }}\left(\frac{\pi Q}{2{Q}_{{{\max }}}}\right),$$

where *E* is excitation energy, *h* is Plank’s constant, *Q* is momentum transfer, and *Q*_max_ is the averaged distance to the edge of the first Brillouin zone, including the effect of the preferred orientation^55,60^. To obtain the *v*_p_ from the IXS spectra, the dispersion was fit with the above sine equation^5–10,16,25,29,33,46,47,50,55,57^. A weighted least-squares method was used with *v*_p_ and *Q*_max_ as free parameters.

### Estimation of sound velocities (*v*_p_ and *v*_s_) at high pressure and temperature

The *v*_p_ of iron, iron alloys, and iron‒light element compounds at high pressure is interpolated/extrapolated by a linear relation (Birch’s law^2^) as

4 $${v}_{{{{{{\rm{p}}}}}}}=A\rho {-}B$$

from present and previous wave velocity measurements: hcp-iron (this study), Fe_0.89_Si_0.11_ (6 wt% silicon)^25^, Fe_0.86_Ni_0.14_ (15 wt% nickel)^27^, Fe_3_S^29^, Fe_7_C_3_^45^, Fe_3_C^46^, FeH^47^, and FeO^50^. The *v*_p_ of iron at high temperature is parameterised by using high temperature data^6,8,16^ combining the present new Birch’s law of hcp-iron at ambient temperature with the following form^16^:

5 $${v}_{{{{{{\rm{p}}}}}}}=A\rho -B-C(T-{T}_{0})\,(\rho_{{{\rm{x}}}}-\rho ),$$

where *A*, *B*, *C*, and *ρ*_x_ are the parameters of the relation, *T* is temperature, and *T*_0_ is ambient temperature, i.e., 300 K. We use the temperature dependency measured experimentally for Fe_0.89_Si_0.11_^22^, Fe_7_C_3_^45^, Fe_3_C^46^, and FeO^50^. The temperature dependency of pure iron is used in Fe_0.86_Ni_0.14_^27^, Fe_3_S^29^, and FeH^47^ as their direct temperature dependencies were not available. Thus, it should be noted that estimations of temperature dependency of those compounds are less reliable.

The *v*_s_ of iron, iron alloys, and iron‒light element compounds are derived by using present and previous wave velocity measurements and EoSs: hcp-iron^3,12^, Fe_0.89_Si_0.11_^25,26^, Fe_0.86_Ni_0.14_^27,28^, Fe_3_S^29,30^, Fe_7_C_3_^45^, Fe_3_C^46^, FeH^47–49^, and FeO^50^. *v*_s_ is related to *v*_p_ and the adiabatic bulk modulus (*K*_*S*_) as follows:

6 $${K}_{S}=\rho \left({v}_{{{{{{\rm{p}}}}}}}^{2}-\frac{4}{3}{v}_{{{{{{\rm{s}}}}}}}^{2}\right).$$

Also, *K*_*S*_ and the isothermal bulk modulus (*K*_*T*_) are defined as follows:

7 $${K}_{T}={K}_{S}-\rho {\gamma }^{2}{c}_{V}T,$$

where *ρ* is the density, *γ* is the Grüneisen parameter, and *c*_*V*_ is the molar heat capacity at constant volume. *c*_*V*_ is derived from the derivative of the molar internal energy (or the pressure) with respect to the temperature,

8 $${c}_{V}={\left(\frac{\partial U}{\partial T}\right)}_{V}=\frac{V}{\gamma }{\left(\frac{\partial P}{\partial T}\right)}_{V},$$

where *U* is the molar internal energy, and *V* is the molar volume^60^. The *P*‒*V*‒*T* relations and the thermodynamic Grüneisen parameter *γ* were given by the EoSs of the alloys and compounds^3,4,26,28,30,45,46,48,50^ with Mie‒Grüneisen‒Debye model. *c*_*V*_ and *K*_*T*_ ‒*K*_*S*_ conversion are derived from above thermodynamic relations with each EoS parameters.

### Differences due to pressure evaluations by different equations of state

Pressure value depends on the EoS (e.g., present maximum experimental density of 13.87 g cm^−3^ correspond to 310 GPa^3^, 314 GPa^4^, and 327 GPa^12^), thus, we need to consider the differences due to EoS. To consider the effects of pressure scales, we compare *ρ*, *v*_p_, and *v*_s_ deficits and amounts of light elements by using another iron EoS of Smith et al. (Smith-EoS)^12^ to the present results based on the EoS of Deweale et al. (Dewaele-EoS)^3^, because pressures by Smith-EoS have larger discrepancies than those evaluated by the EoS of Fei et al.^4^. Supplementary Fig. 6 shows the pressure dependence of *v*_p_ of hcp-iron at ambient temperature, in which pressures are evaluated by the Smith-EoS. Compared to the result by Dewaele-EoS, the maximum experimental pressure was evaluated as 327 GPa, which is 17 GPa higher than that by Dewaele-EoS. As mentioned above, *v*_s_ is derived from *v*_p_ and EoS, Supplementary Fig. 7 shows the *v*_s_‒*ρ* relation of hcp-iron derived by using Smith-EoS. The results with Smith-EoS shows better consistency with theoretical results^6,10^ than that of Dewaele-EoS, however, both do not show significant deviations and are within the uncertainty. Supplementary Fig. 8 shows the *v*_p_‒*ρ* and *v*_s_‒*ρ* relations for pure hcp-iron at ICB and COE depending on the Smith-EoS and those of the PREM inner core. The *v*_p_ and *v*_s_ deficits by Smith-EoS are 1(±1)% and 24(±16)% at ICB and 4(±2)% and 29(±15)% at COE, respectively. Therefore, we conclude that there are no significant deviations in the discussions of *v*_p_ and *v*_s_ deficits.

## Supplementary information

Supplementary Information

## Data Availability

All data used in this study are presented in the text, Methods, and supplementary information. Experimental conditions and results are summarised in Table 1. The data used to produce figures can be accessed from the public repository Zenodo.

## Code availability

We used IPAnalyzer/PDindexer/ReciPro software package^58,59^, which is available at https://github.com/seto77/.
