# Supplementary material for: Sound velocity of hexagonal close-packed iron to the Earth’s inner core pressure
Source: Nat Commun. 2022 Nov 25;13:7211. doi: 10.1038/s41467-022-34789-2 (PMC9700692; doi:10.1038/s41467-022-34789-2)
Supplement: Supplementary file 1 — Supplementary Information [file 41467_2022_34789_MOESM1_ESM.pdf]

Supplementary Information

Sound velocity of hexagonal close-packed iron to the Earth's inner core pressure

Daijo Ikuta*, Eiji Ohtani*, Hiroshi Fukui, Takeshi Sakai, Daisuke Ishikawa, and Alfred Q. R. Baron*

*Corresponding authors. Email: dikuta@tohoku.ac.jp, eohtani@tohoku.ac.jp, and baron@spring8.or.jp

This PDF file includes:

Supplementary Figs.: 1–8
Supplementary References: 1–25

Creative Commons Attribution 4.0 International License (CC BY 4.0)

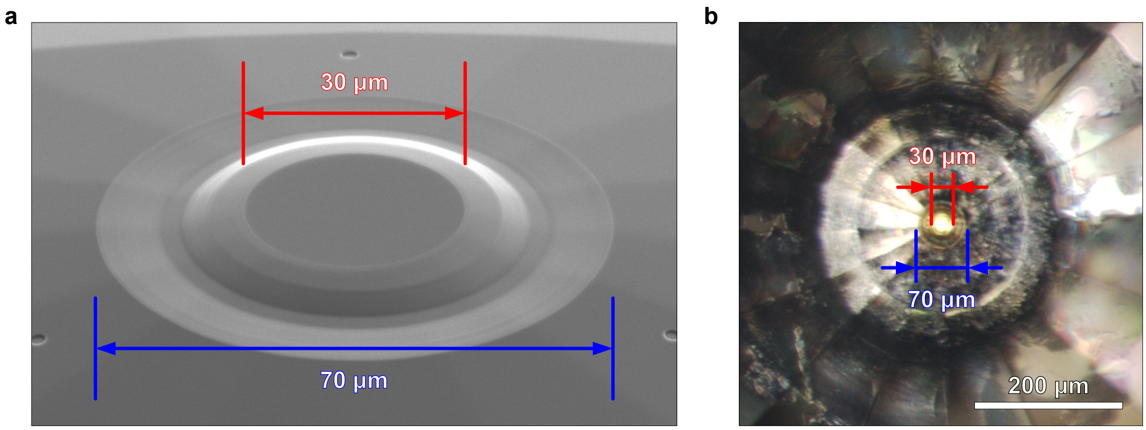

Supplementary Fig. 1. The stepped bevel diamond anvil. **a** Scanning electron microscope (SEM) image of the stepped bevel diamond anvil after machining by a dual focused ion beam (FIB). The red and blue arrows represent the 30 μm culet and a flat surface of 70 μm in diameter with a depth of ~3 μm outside the 30 μm culet of (100)-oriented double bevel anvils, respectively (see also Supplementary Fig. 2). **b** Optical microscope image at highest experimental pressure condition (310–327 GPa[1–3]). The coloured arrows are the same as (**a**). The brightest area (~20 μm) inside the 30 μm cullet is the iron sample.

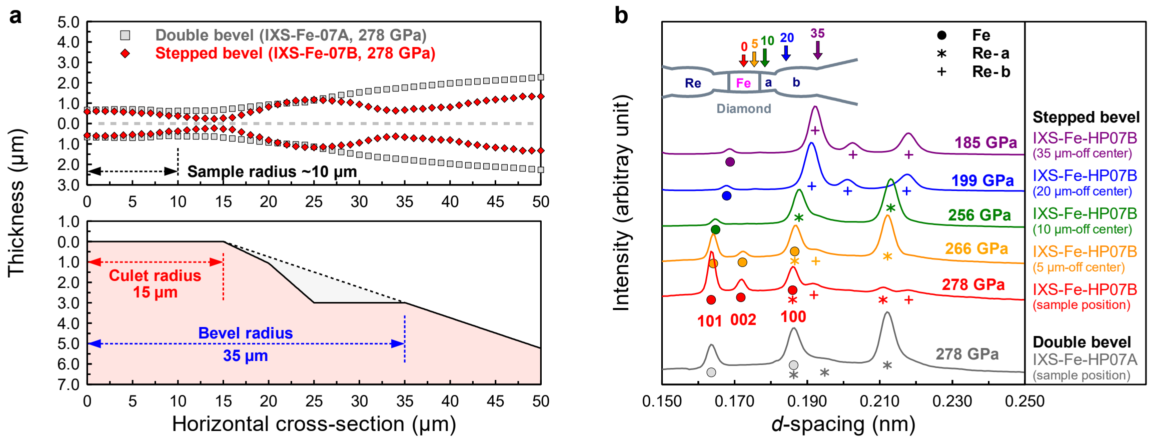

Supplementary Fig. 2. High-pressure generation in a stepped bevel anvil. **a** Upper column shows that sample/rhenium gasket thickness distribution with a stepped bevel anvil (red diamonds) and a double bevel anvil (grey squares) at 278 GPa[1] derived from x-ray absorption measurements. The grey dashed line represents the centre of the sample/rhenium gasket. The bottom column shows the schematic diagram representing the initial shape of both stepped and double bevel anvils. The red shaded area represents the stepped bevel anvil and grey shaded area indicates the part machined by FIB from the initial double bevel anvil. **b** Integrated x-ray diffraction (XRD) profiles at 278 GPa[1] with a stepped bevel anvil in sample position (red), and 5 (yellow), 10 (green), 20 (blue), and 35 (purple) μm off-centred positions. The position of each XRD pattern is shown schematically in the upper left. The grey line shows the integrated XRD patterns at a sample position at 278 GPa[1] with a double bevel anvil. The pressures in each position derived from the hexagonal close-packed (hcp) iron peaks based on Dewaele-EoS[1] are also shown. The circle and asterisk symbols represent the peaks of hcp-iron and rhenium, respectively. Re-a and Re-b are inferred to be the peaks from the rhenium gasket contacted with the diamond culet and the gasket outside the diamond culet, respectively. Those positions are also indicated in the upper left.

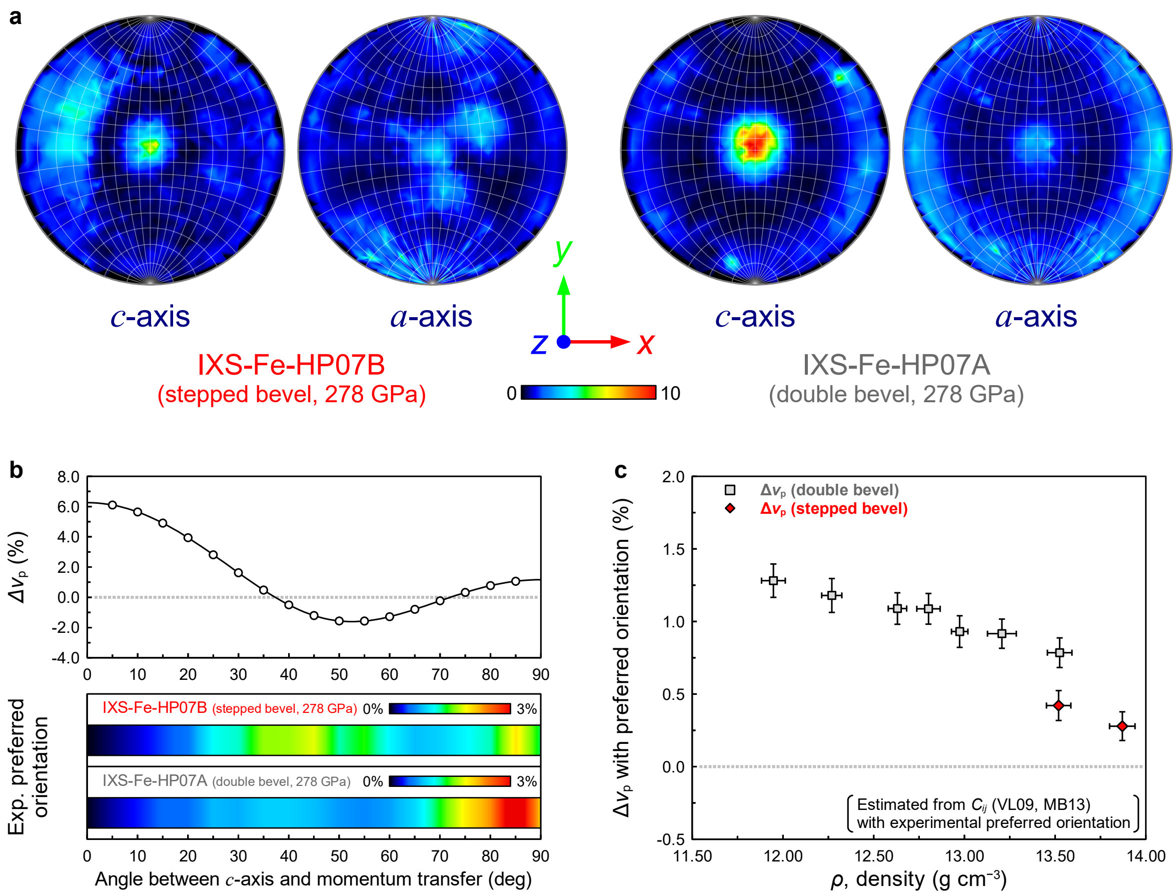

Supplementary Fig. 3. Estimation of the anisotropy of the velocity in the experimental conditions. **a** The experimental probability densities for *c*- and *a*-axis concentrations of hcp-iron in both stepped and double bevel anvil experiments at 278 GPa[1] with stereographic projections, derived from the XRD patterns. The *x*-*y*-*z* arrows represent each direction of the experimental apparatus; *x*: horizontal, *y*: vertical, and *z*: compression directions of a diamond anvil cell (DAC), respectively. The colour scale bar represents the multiple of uniform density. For stepped bevel anvil experiment, we observed the preferred orientation that the *c*-axis in all crystals grains has been concentrated about six times around the compression direction compared with a random orientation. On the other hand, for double bevel anvil experiment, the *c*-axis has been much more concentrated, about ten times. Although the *a*-axis was also unevenly distributed, but the concentration was weak compared to the *c*-axis. **b** Upper panel shows $\Delta v_p(\psi)$ $\{= [v(\psi)-v_{VRH}]/v_{VRH}\}$ as a function of angle ψ (where ψ is the angle between *c*-axis and the lattice vibration direction due to inelastic x-ray scattering) at 13.52 g cm^{-3} (corresponding to 278 GPa[1]), calculated using the elastic constants, C_{ij}, from theoretical calculations (VL09, MB13)[4,5]. The v_{VRH} is the average velocity of Voigt–Reuss–Hill average (VRH) obtained from C_{ij}. (e.g., ref. 6). The open circles with curves represent velocity anisotropies compared with the v_p of VRH. The lower panel shows the probability density of the grains with the preferred orientation as a function of ψ for both stepped bevel anvil and double bevel anvil experiments at 278 GPa[1]. The probability densities were shown as one-dimension as a function of ψ by integrating (**a**). The colour contour represents the concentrations of crystal directions in the experimental grains. While the stepped bevel anvil experiment had widely distributed preferred orientation at 30–90 degrees, the double bevel anvil experiment has strong preferred orientation concentrated at ~85 degrees. **c** Δv_p for both stepped bevel and double bevel diamond anvil experiments as a function of density. Δv_p is the integrated velocity difference weighted by the preferred orientation, which is derived from the integration, $\frac{1}{\pi}\int_0^{\pi} x(\psi)\Delta v_p(\psi)\,d\psi$, where $x(\psi)$ is the weight derived from the preferred orientation [here, $x(\psi)$ is normalised as $\frac{1}{\pi}\int_0^{\pi} x(\psi)\,d\psi = 1$. Red diamonds are the anisotropies in stepped bevel anvil experiments and grey squares are those in double bevel anvil experiments. The magnitudes of anisotropy on v_p are <0.5% and <1.3% for stepped bevel anvil and double bevel anvil experiments, respectively. The error bars represent 1σ uncertainties. The effect of anisotropy on v_p is within the uncertainty of the present v_p measurement shown in Table 1.

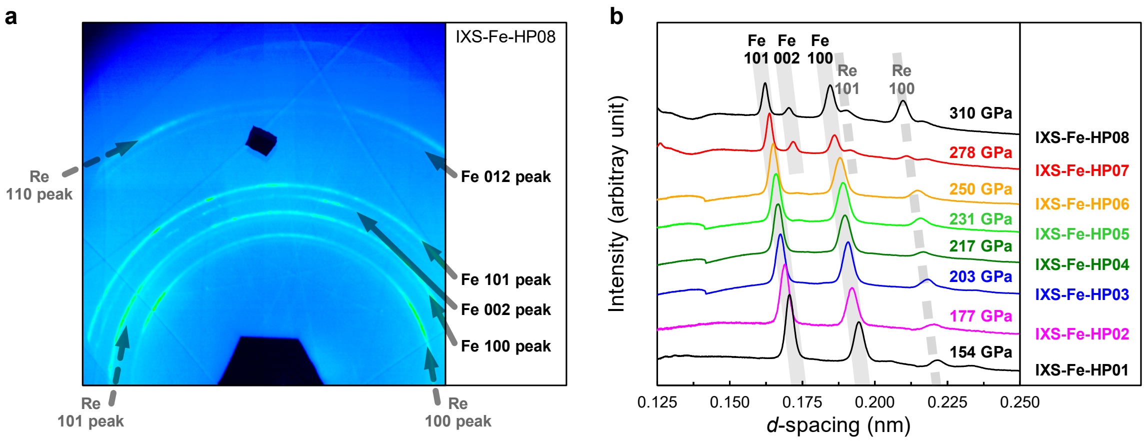

Supplementary Fig. 4. XRD image and integrated patterns. **a** XRD image at 13.87 g cm^{-3} (corresponding to 310 GPa[1]). Typical hcp-iron (sample) and rhenium (gasket) peaks are indicated by arrows. The black areas are lead shadows for detector protection. **b** Integrated XRD patterns at all experimental pressures. The pressures in each experiment derived from the hcp-iron peaks based on Dewaele-EoS[1] are also shown. Bold and dashed lines represent the change of hcp-iron and rhenium peak positions, respectively.

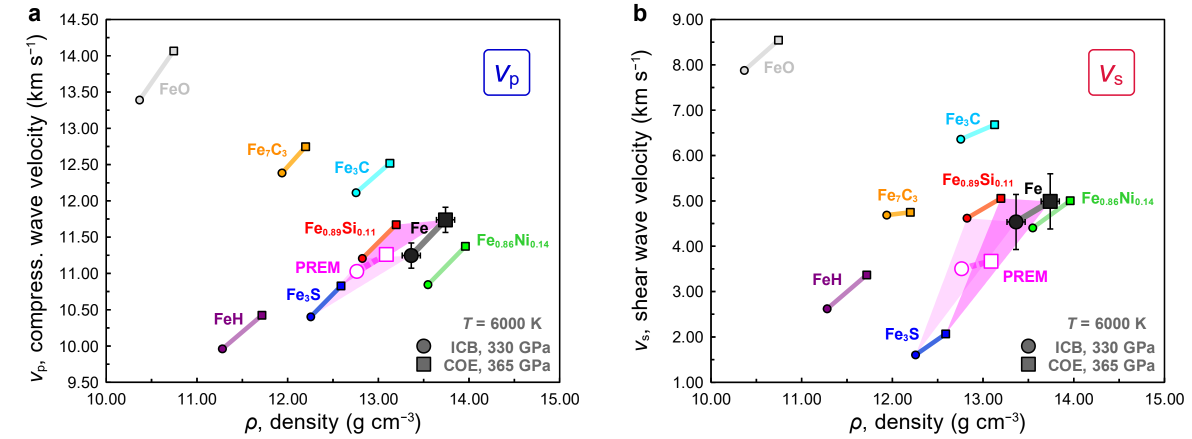

Supplementary Fig. 5. Comparison of v–ρ relations of hcp-iron, iron alloys, and iron compounds with Preliminary reference Earth model (PREM) at the inner core boundary (ICB, 330 GPa, and 6000 K) and centre of the Earth/core (COE, 365 GPa, and 6000 K) conditions, (**a**) v_p–ρ relations of each material, (**b**) v_s–ρ relations of each material. The circle and square symbols indicate ICB and COE conditions, respectively. The coloured symbols are as follows: black is hcp-iron by this study (pressure is evaluated by the Dewaele et al.[1]), red is $Fe_{0.89}Si_{0.11}$ (6 wt% silicon, hcp)[7,8], green is $Fe_{0.86}Ni_{0.14}$ (15 wt% nickel, hcp)[9,10], blue is Fe_3S (tetragonal)[11,12], orange is Fe_7C_3 (orthorhombic)[13], cyan is Fe_3C (non-magnetic, orthorhombic)[14], purple is FeH (dhcp)[15–17], grey is FeO (cubic B1)[18], and magenta (open symbol) is PREM[19]. The v–ρ relations for hcp-iron at the ICB and COE conditions are determined by this work (see text and Methods). The v–ρ relations of other iron alloys and iron compounds are calculated from previous studies on sound velocity and EoSs[7–18], and the present temperature dependence of hcp-iron (details are in Methods) except for $Fe_{0.89}Si_{0.11}$, Fe_7C_3, Fe_3C, and FeO in which the temperature dependencies were experimentally determined[7,13,14,18]. The magenta triangles represent the potential v–ρ relations of the iron compound consisting of three endmembers (hcp-iron, $Fe_{0.89}Si_{0.11}$, and Fe_3S) (lighter and darker colours indicate ICB and COE conditions, respectively). The error bars represent 1σ uncertainties for hcp-iron.

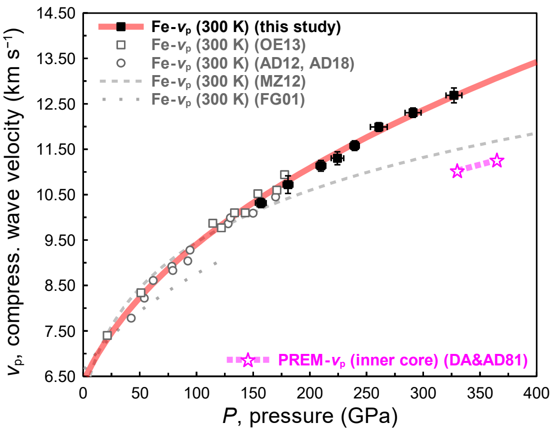

Supplementary Fig. 6. The pressure dependence of v_p of hcp-iron at ambient temperature. The pressure was evaluated by the isothermal EoS of hcp-iron (ρ_0 = 8.45 g cm^{-3}, K_{T0} = 177.5 GPa, K'_{T0} = 5.66) derived from the isentrope of hcp iron by Smith-EoS[3]. Solid squares are the results of this work; open squares from Ohtani et al. (OE13)[20]; circles from Antonangeli et al. (AD12, AD18)[21,22]; a dashed curve extrapolated by a power-law relation from Mao et al. (MZ12)[23]; and a dotted curve from Fiquet et al. (FG01)[24,25]. The magenta dashed curve with stars is the PREM inner core (DA&AD81)[19]. The symbols are the same as those in Fig. 2. The error bars represent 1σ uncertainties.

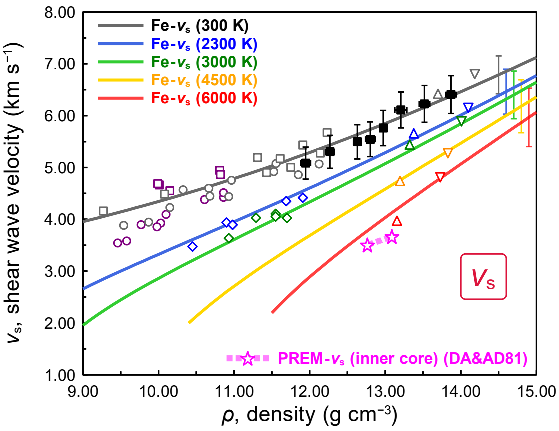

Supplementary Fig. 7. v_s–ρ relations using Smith-EoS[3]. We used the same experimental and theoretical data as those given in Fig. 3. The error bars represent 1σ uncertainties.

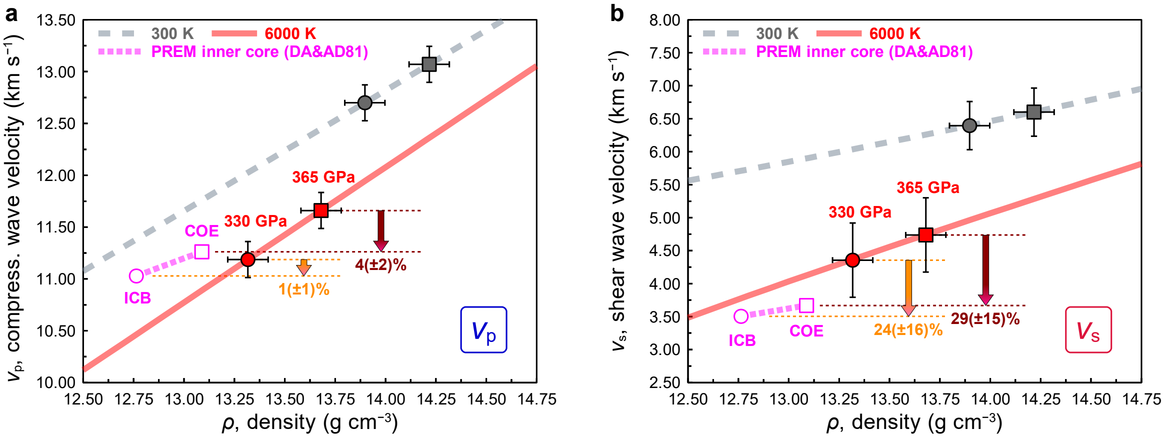

Supplementary Fig. 8. Comparison of density–velocity relations of hcp-iron based on Smith-EoS[3] at inner core conditions with PREM. The v_p deficit (**a**) and the v_s deficit (**b**) in the inner core. The symbols are the same as those in Fig. 4. The v_p deficit and the v_s deficit are 1(±1)% and 24(±16)% at ICB, and 4(±2)% and 29(±15)% at COE, respectively. The error bars represent 1σ uncertainties.

Supplementary References

1. Dewaele, A. et al. Quasihydrostatic equation of state of iron above 2 Mbar. Phys. Rev. Lett. 97, 215504 (2006). https://doi.org/10.1103/PhysRevLett.97.215504
2. Fei, Y., Murphy, C., Shibazaki, Y., Shahar, A. & Huang, H. Thermal equation of state of hcp-iron: Constraint on the density deficit of Earth's solid inner core. *Geophys. Res. Lett.* **43**, 6837–6843 (2016). https://doi.org/10.1002/2016GL069456
3. Smith, R. F. et al. Equation of state of iron under core conditions of large rocky exoplanets. *Nat. Astron.* **2**, 452–458 (2018). https://doi.org/10.1038/s41550-018-0437-9
4. Vocadlo, L., Dobson, D. P. & Wood, I. G. Ab initio calculations of the elasticity of hcp-Fe as a function of temperature at inner-core pressure. *Earth. Planet. Sci. Lett.* **288**, 534–538 (2009). https://doi.org/10.1016/j.epsl.2009.10.015
5. Martorell, B., Vocadlo, L., Brodholt, J. & Wood, I. G. Strong premelting effect in the elastic properties of hcp-Fe under inner-core conditions. *Science* **342**, 466-468 (2013). https://doi.org/10.1126/science.1243651
6. Poirier, J. P. *Introduction to the physics of the Earth's interior 2nd edition* (Cambridge University Press, 2000). https://doi.org/10.1017/CBO9781139164467
7. Sakairi, T. et al. Sound velocity measurements of hcp Fe-Si alloy at high pressure and high temperature by inelastic x-ray scattering. *Am. Mineral.* **103**, 85–90 (2018). https://doi.org/10.2138/am-2018-6072
8. Edmund, E. et al. Velocity-density systematics of Fe-5wt%Si: Constraints on Si content in the Earth's inner core. *J. Geophys. Res.* **124**, 3436–3447 (2019). https://doi.org/10.1029/2018JB016904
9. Wakamatsu, T., Ohta, K., Yagi, T., Hirose, K. & Ohishi, Y. Measurements of sound velocity in iron-nickel alloys by femtosecond laser pulses in a diamond anvil cell. *Phys. Chem. Miner.* **45**, 589–595 (2018). https://doi.org/10.1007/s00269-018-0944-3
10. Sakai, T. et al. Equation of state of pure iron and $Fe_{0.9}Ni_{0.1}$ alloy up to 3 Mbar. *Phys. Earth Planet. Inter.* **228**, 114–126 (2014). https://doi.org/10.1016/j.pepi.2013.12.010
11. Kamada, S. et al. The sound velocity measurements of Fe_3S. *Am. Mineral.* **99**, 98–101 (2014). https://doi.org/10.2138/am.2014.4463
12. Kamada, S. et al. Equation of state of Fe_3S at room temperature up to 2 megabars. *Phys. Earth Planet. Inter.* **228**, 106–113 (2014). https://doi.org/10.1016/j.pepi.2013.11.001
13. Prescher, C. et al. High Poisson's ratio of Earth's inner core explained by carbon alloying. *Nat. Geosci.* **8**, 220–223 (2015). https://doi.org/10.1038/ngeo2370
14. Takahashi, S. et al. Sound velocity of Fe_3C at high pressure and high temperature determined by inelastic x-ray scattering. *C. R. Geosci.* **351**, 190–196 (2019). https://doi.org/10.1016/j.crte.2018.09.005
15. Shibazaki, Y. et al. Sound velocity measurements in dhcp-FeH up to 70 GPa with inelastic x-ray scattering: Implications for the composition of the Earth's core. *Earth Planet. Sci. Lett.* **313–314**, 79–85 (2012). https://doi.org/10.1016/j.epsl.2011.11.002
16. Sakamaki, K. et al. Melting phase relation of FeH_x up to 20 GPa: Implication for the temperature of the Earth's core. *Phys. Earth Planet. Inter.* **174**, 192–201 (2009). https://doi.org/10.1016/j.pepi.2008.05.017
17. Kato, C. et al. Stability of fcc phase FeH to 137 GPa. *Am. Mineral.* **105**, 917–921 (2020). https://doi.org/10.2138/am-2020-7153

18. Tanaka, R. et al. The sound velocity of wustite at high pressures: Implications for low-velocity anomalies at the base of the lower mantle. *Prog. Earth Planet. Sci.* **7**, 23 (2020). https://doi.org/10.1186/s40645-020-00333-3

19. Dziewonski, A. M. & Anderson, D. L. Preliminary reference Earth model. *Phys. Earth Planet. Inter.* **25**, 297–356 (1981). https://doi.org/10.1016/0031-9201(81)90046-7

20. Ohtani, E. et al. Sound velocity of hexagonal close-packed iron up to core pressures. *Geophys. Res. Lett.* **40**, 5089–5094 (2013). https://doi.org/10.1002/grl.50992

21. Antonangeli, D. et al. Simultaneous sound velocity and density measurements of hcp iron up to 93 GPa and 1100 K: An experimental test of the Birch's law at high temperature. *Earth Planet. Sci. Lett.* **331–332**, 210–214 (2012). https://doi.org/10.1016/j.epsl.2012.03.024

22. Antonangeli, D. et al. Sound velocities and density measurements of solid hcp-Fe and hcp-Fe-Si (9 wt.%) alloy at high pressure: Constraints on the Si abundance in the Earth's inner core. *Earth Planet. Sci. Lett.* **482**, 446–453 (2018). https://doi.org/10.1016/j.epsl.2017.11.043

23. Mao, Z. et al. Sound velocities of Fe and Fe-Si alloy in the Earth's core. *Proc. Natl. Acad. Sci. USA.* **109**, 10239–10244 (2012). https://doi.org/10.1073/pnas.1207086109

24. Fiquet, G., Badro, J., Guyot, F., Requardt, H. & Krisch. M. Sound velocities in iron to 110 gigapascals. *Science* **291**, 468–471 (2001). https://doi.org/10.1126/science.291.5503.468

25. Antonangeli, D. & Ohtani, E. Sound velocity of hcp-Fe at high pressure: Experimental constraints, extrapolations and comparison with seismic models. *Prog. Earth Planet. Sci.* **2**, 3 (2015). https://doi.org/10.1186/s40645-015-0034-9
